# Torsional Ultrasound Sensor Optimization for Soft Tissue Characterization

**DOI:** 10.3390/s17061402

**Published:** 2017-06-15

**Authors:** Juan Melchor, Rafael Muñoz, Guillermo Rus

**Affiliations:** Department of Structural Mechanics, University of Granada; ETS Ingenieros de Caminos, Severo Ochoa s/n, 18071 Granada, Spain; rmb@ugr.es (R.M.); grus@ugr.es (G.R.)

**Keywords:** torsional ultrasound, probability of detection, soft tissue mechanics, finite element method, optimization, inverse problem

## Abstract

Torsion mechanical waves have the capability to characterize shear stiffness moduli of soft tissue. Under this hypothesis, a computational methodology is proposed to design and optimize a piezoelectrics-based transmitter and receiver to generate and measure the response of torsional ultrasonic waves. The procedure employed is divided into two steps: (i) a finite element method (FEM) is developed to obtain a transmitted and received waveform as well as a resonance frequency of a previous geometry validated with a semi-analytical simplified model and (ii) a probabilistic optimality criteria of the design based on inverse problem from the estimation of robust probability of detection (RPOD) to maximize the detection of the pathology defined in terms of changes of shear stiffness. This study collects different options of design in two separated models, in transmission and contact, respectively. The main contribution of this work describes a framework to establish such as forward, inverse and optimization procedures to choose a set of appropriate parameters of a transducer. This methodological framework may be generalizable for other different applications.

## 1. Introduction

Torsional ultrasonic waves can be used to characterize the biomechanics of soft tissue. The design process of a torsional ultrasonic sensor has as a key point the use of piezoelectric materials [1,2]. Piezoelectric materials (PZT) are an inorganic compound that changes shape when an electric field is induced. They are actually used in the design of sensors in several applications, such as music microphones and instruments, precision positioning, cancellation of noise, motors, and ultrasonic devices in our study.

This design problem provides two steps, the understanding of propagation of torsional waves and the optimization of a transducer with the capacity to transmit and receive them. Additionally, some applications require large displacements, such as improvement of micropositioning in robotics, CD drivers, or control of trailing edge flaps of helicopters. For the rest, these transducers are able to execute large circumferential displacements, it is necessary for smart structures, and they can be adapted for a range of applications on a wide variety of requirements [3,4].

Several optimization criteria are available for ultrasonic transducers depending on specific parameters being considered. For example, the use of central frequency and insertion loss (a factor associated with frequencies that disappear through a filter). Furthermore, new variables as the case of central frequency or the energy have been shown to be the most appropriate. Other new features have been introduced that describe the waveform and amplitude spectra improving the inputs of the optimization formulation, widening the range of applications as it is shown in this work [5,6].

The combined use of simulation based on finite elements analysis (FEA) and optimization methods leads to a suitable way of designing ultrasonic sensors. This process needs the use of a global cost function that measures the maximization/minimization criterion and is calculated using the model. Several design alternatives can be obtained using different parameters to optimize the cost function [7,8].

The use of FEA together with the multi-objective decision facilitates an efficient design where conflicts among several criteria such as acoustic effects, impedance, vibration of the sensors are optimized, in addition to the design time [9,10].

Recently, sensors which can be used for similar applications have been designed. Bolt-Langevin type transducers (BLT) are an example, composed of piezoelectric disks with a pair of elastic bodies mounted at the ends which generate mechanical oscillations with a specific design frequency. They mainly use longitudinal waves in the thickness direction. Additionally, there are some works taking into account the torsional vibrations generated by the elastic part [11,12,13,14]. The main application of this type of sensors was the generation of uniform micro-droplets [15], as well as in acoustic levitation (NFAL), in non-destructive testing, and biomedical engineering [16,17].

Numerical analysis may be useful to consider other designs, setting the tolerance of each parameter related to sensor efficiency within a particular range [18,19].

Torsional waves has also been used as guided waves in nondestructive testing of pipes [20,21], in liquids inserting a bar in the fluid where waves are propagated to measure density [22,23], liquid level [24], temperature [24], or viscosity [23].

One of the main applications of ultrasonic transducers in medicine is elastography with ultrasonic longitudinal waves, where there exists a special interest in efficiency enhancements of sensor desings [25,26,27,28]. Recently, a new field of investigation has been opened introducing the concept of transversal waves to measure the shear modulus of soft tissue, due to the importance of correlating it with many disorders [29,30].

Accordingly, the motivation of this study began with the aim at designing an optimized ultrasonic sensor focus on clinical applications to obtain high levels of sensitivity in mechanical identification of soft tissue. A part of the computational design of the transducer was described in detail [31]. In parallel to this, since the first design study, some reference works have emerged as another shear ultrasonic sensor based on induced resonances [32] providing a new screening method for breast cancer from wave viscoelasticity imaging [33]. The last approaches in this line of research have improved developing the generation of remote adaptive torsional shear waves (ATSW) using an octagonal phased array to improve displacements and reducing the dispersion of shear wave speeds. The ATSW method also reveal that it is possible to estimate the viscoelasticity of biological tissues when small biases in lesion appear [34,35]. Torsional waves has also been used to characterized mechanical properties of tissues like liver [36] or vocal fold [37,38].

The transducer is going to be adapted at facing preterm birth assessment in direct contact with cervix, since the survival and morbidity carries a burden in the health care system [39]. The prevalence of preterm birth is between 8.1–12.7% of all births in European countries [40].

The concept of shear waves to evaluate cervix stiffness to birth prediction has been considered with another physical principle, Supersonic Shear Imaging (SSI), without separation between P and S waves and high energy levels, since shear waves are generated using the nonlinear acoustic radiation force [29,41]. On the other hand, direct contact can be avoided.

This paper describes the complete view of the methodology that has been used to design the torsinal wave transducer for soft tissue evaluation in direct contact, dividing it in two phases. The first one studies only the torsional propagation in tissue and how the excitation conditions are optimized using an inverse problem. It is based on a finite element model (FEM) of the tissue by FEAP open software. The second phase optimize the transducer design adding the FEM of the transducer and using a inverse problem that maximize, what the authors call the robust probability of detection (RPOD) [31], as a measure of the sensibility of the transducer. The probability of detection (POD) [42] of the transducer as a function of a model parameter measures the probability of the transducer to detect a specific small change in that parameter. The RPOD concept is introduced to deal with more than one parameter, conservatively selecting the worse POD of the parameters as the representative POD of the transducer. This definition of RPOD will be used in an inversion procedure to optimize the parameters of the transducer that maximize the RPOD.

## 2. Methods

The optimization of the proposed design of the torsional wave transducer has been undertaken in two phases:

- Phase 1: Modelization of the torsional wave propagation in tissue, including a sensitivity test on the parameters of the excitation wave.
- Phase 2: Joint modelization of tissue-transducer interaction. Optimization of the transducer under maximum POD (Probability Of Detection) criterion.

The purpose of phase 1 is to test the capacity of the torsional wave model and the inverse method to identify changes in mechanical properties of a multilayered tissue, independently on the transducer that is used. Additionally, the POD estimator is checked as the tool to measure sensitivity. Phase 2 incorporates the modelization of the proposed transducer design and uses the POD estimator to optimize the transducer design maximizing sensitivity measure. Both phases use a similar (not identical) inverse problem design. The common parts of the inverse problem and POD are firstly presented, and afterwards the phases.

**Figure 1:**
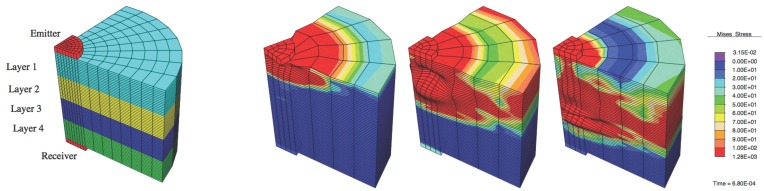
Left: geometry, layers, transducers and mesh. Following pictures: three instants of torsional propagation *t*= 280, 460 and 680 μs, using a simpler mesh.

**Figure 2:**
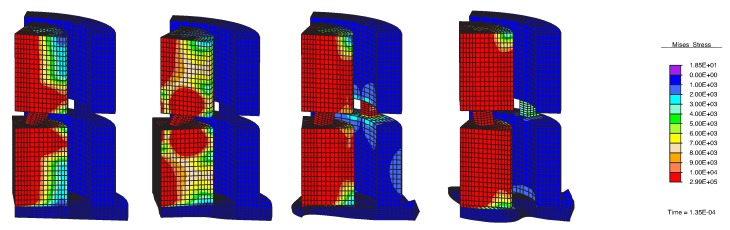
Torsional transducer at instants *t* = 9, 18, 117, 135 μs. Tissue is the soil.

In this work, POD is the algorithm to measure sensitivity and may be defined as the probability of the transducer and the reconstruction algorithm to detect changes on the mechanical properties of the tissue, given the presence of uncertainties, mainly, signal noise. A multilayered tissue will be supposed, four layers in phase 1 (Figure 1) just to test the multilayer capacity, and two layers in phase 2 (dermic and connective, Figure 2). The mechanical parameters are Young Modulus and Reynolds attenuation in phase 1 for each layer, and shear moduli for both layers of phase 2.

### 2.1. Robust Probability of Detection

A plausible pathology may be inferred when enough changes in the mechanical properties of the tissue layers are detected. Let **P** be a specific parametrization for POD study, consisting of a vector with the mechanical properties under study. For instance for phase 2 shear moduli *G*, $P=(G_{c}\;G_{d})$ (*c* connective and *d* dermic). A pathological change implies that the healthy state presents values of reference *r* on the parameters, and pathology would exist in case that enough variation is observed $p=\left(\Delta G_{c}\;\Delta G_{d}\right)$. If $P_{k}$ is a generic element of the array **P**, $p_{k}=\Delta P_{k}=P_{k}-P_{k}^{r}$, being $N_{p}$ the size of the array.

The mathematical definition of the probability of detection (POD) is presented as the probability that the modifications on the received signal (signal) given the emergence of pathology surpasses the signal noise level (noise),

(1) $$\mathrm{POD}\;=\;P\left(\;\frac{\left|\;\mathrm{SIGNAL}\;\right|{\;}^{2}}{\left|\;\mathrm{NOISE}\;\right|{\;}^{2}}>1\;\right)$$

The variables that determines the POD estimation for a specific location are the severity of the pathology (**P**) and the noise level *σ*. The following deduction will be shown for one single parameter $p_{k}$, considering that this calculation will have to be repeated $N_{p}$ times, one for each parameter of **p**.

As signal and noise changes depend on several parameters $p_{k}$, a robust POD (RPOD), is defined so that the RPOD is the POD of the less favorable case, the POD of the parameter with worst POD.

(2) $$\mathrm{RPOD}=\min_{k}\mathrm{POD}(p_{k})\;\;\mathrm{POD}\left(p_{k}\right)=P\left(\frac{{\left|\mathrm{SIGNAL}(p_{k})\right|}^{2}}{{\left|\mathrm{NOISE}(p_{k})\right|}^{2}}>1\right)$$

The linear nature of the physical models that will be used, assumes a linear relation between measurements and mechanical properties. The measurement dependency with noise will also be suppose to be linear. Under these two assumptions of linearity, the measurements may be developed as a Taylor expansion of first order, centered at the healthy case and the absence of noise.

(3) $$\psi_{i}\left(p_{k},\sigma \right)=\psi_{i}\left(0,0\right)+\underset{\mathrm{SIGNAL}}{\underset{︸}{p_{k}\frac{\partial \psi_{i}}{\partial p_{k}}\left(0,0\right)}}+\underset{\mathrm{NOISE}}{\underset{︸}{\sigma \frac{\partial \psi_{i}}{\partial \sigma }\left(0,0\right)}}+hot$$

being i=1,…,N the receiving points. $\psi_{i}\left(0,0\right)$ are the measurements in the absence of pathology and noise at point *i*, the second term the linear variation of the measurement given the only presence of the pathology, which has been labeled as signal, and the third term the linear variation due to the presence of noise and healthy conditions, labeled noise.

The second term of the sum may be approximately expressed as a finite difference, but introducing a very small degradation $p_{k0}\to 0$ instead of a pure healthy state to assure that the FEM computational implementation of the models captures the perturbations produced at small $p_{k}$, and compute $\psi_{i_{,p_{k}}}\left(p_{k0},0\right)\approx \psi_{i_{,p_{k}}}\left(0,0\right)$,

$$\frac{\partial \psi_{i}}{\partial p_{k}}\left(p_{k0},0\right)=\psi_{i_{,p_{k}}}\left(p_{k0},0\right)=\frac{\psi_{i}\left(p_{k0}+\Delta p_{k},0\right)-\psi_{i}\left(p_{k0},0\right)}{\Delta p_{k}}$$

For the RPOD calculation, the FEM algorithm must be run $N_{p}+1$ times. The first one, to calculate the signal applying the mechanical properties of reference (representing no degradation), $\psi_{i}\left(p_{k0},0\right)$, and $N_{p}$ additional signals altering each parameter of the parametrization, $\psi_{i_{,p_{k}}}\left(p_{k0}+\Delta p_{k},0\right)$.

The noise term of the *Taylor* series on Equation (3) is proposed to be,

(4) $$\frac{\partial \psi_{i}}{\partial \sigma }=\xi_{i}\mathrm{RMS}\left(\psi_{i}^{\mathrm{FEM}}\right)=\xi_{i}\mathrm{RMS}\;\;\;\mathrm{RMS}\left(g\right)=\sqrt{\frac{1}{M}\sum_{j=0}^{M-1}g{\left(t_{j}\right)}^{2}}$$

with $\xi_{i}$ a random variable (noise generator) applied over the root mean square (RMS) of the simulated signal. (RMS) defined for any sampled function *g* in time domain $g(t_{j})$ at *M* samples.

Equations (3) and (4), and the relationship ${\left|Y_{i}\right|}^{2}=\frac{1}{m}\sum_{i=1}^{m}Y_{i}^{2}$, can be applied on Equation (1),

(5) $$\begin{array}{cc} \mathrm{POD} & =P\left(\frac{p_{k}^{2}\frac{1}{N}\sum_{i=1}^{N}{\left(\psi_{i_{,p_{k}}}\left(0,0\right)\right)}^{2}}{\sigma^{2}{\mathrm{RMS}}^{2}\frac{1}{N}\sum_{i=1}^{N}\xi_{i}^{2}}>1\right)=P\left(p_{k}^{2}>\frac{{\mathrm{RMS}}^{2}\sigma^{2}\sum_{i=1}^{N}\xi_{i}^{2}}{S_{k}}\right) \end{array}$$

being,

(6) $$S_{k}=\sum_{i=1}^{N}{\left(\psi_{i_{,p_{k}}}\left(0,0\right)\right)}^{2}$$

The POD may be reinterpreted in terms of parameter noise, so that it is the probability of a specific stochastic mechanical property $p_{k}^{2}$, having the following cumulative probability density function,

(7) $$\mathrm{POD}\;=\;F\left(\frac{{\mathrm{RMS}}^{2}\sigma^{2}\sum_{i=1}^{N}\xi_{i}^{2}}{S_{k}}\right)$$

which represents the noise distribution function in terms of the parameter $p_{k}$ (in this case, a mechanical property of the material). That is, how the noise distribution function of the signal is translated through the direct model into a random variation of the parameter values. In order to detect a variation of the mechanical properties originated by a pathological zone, this variation must exceed this parameter-noise level, a fact that defines the POD.

Following techniques of the theory of error propagation and Monte Carlo sampling, the noise of the signals at receiving locations can be assumed to behave as a normal distribution [42], and consequently, the squared sum of the noise $\xi_{i}$ follows a *chi-square* distribution, given that $\sum_{i=1}^{N}\xi_{i}^{2}⟶\chi_{N}^{2}$ (e.g., [43]). Its parameter is the number *N* of degrees of freedom, corresponding to the number of receiving locations. This *Chi-square* distribution may be approximately assumed as a normal N distribution in the case of N>10, with N−2/3 the mean and $\sqrt{2N}$ the standard deviation, $\chi^{2}\left(N\right)\;\approx \;N\left(N-2/3,\sqrt{2N}\right)$. In (7) it leads to,

(8) $$p_{k}^{2}⟶N\left[\frac{{\mathrm{RMS}}^{2}\;\sigma^{2}\;\left(N-2/3\right)}{S_{k}},\frac{{\mathrm{RMS}}^{2}\;\sigma^{2}\;\sqrt{2N}}{S_{k}}\right]$$

expression that is only valid for noise with Gaussian distribution.

As this probability density function *f* posses a cumulative probability $F\left(x\right)=\int_{-\infty }^{x}f\left(y\right)dy$, and being its inverse x=G(F(x)), the pathology to noise ratio $p_{k}/\sigma$ can be deduced from (8) for a fixed and desired POD value as,

(9) $$\frac{p_{k}}{\sigma }=\sqrt{\frac{{\mathrm{RMS}}^{2}\;\left(N-2/3\right)}{S_{k}}\;\left(1+G\left[\mathrm{POD}\right]\;\frac{\sqrt{2N}}{N-2/3}\right)}$$

This is the final formula, where a specific value of POD can be fixed, to obtain the relationship of the alteration of the mechanical property $p_{k}$ against noise, around the mechanical property’s value of reference.

### 2.2. Inverse Problem

Two different IP approaches has been used. In phase 1 to adjust the mechanical properties of the tissue that minimize the difference between simulated and experimental signals (misfit function), and in phase 2 to optimize the transducer parameters that maximize the RPOD.

Although the target is different, both approaches present similarities. On one hand, Genetic Algorithms (GA) are used, mainly because they are global search algorithms. It implies that the search for the optimal parameters is made by reasonably exploring the whole parameter space, so that falling in local maxima or minima is avoided (see [42,44] for more detailed information on the algoritm that is used). On the other hand, the cost functional will serve the optimization criterion. If *f* is the function to optimize (misfit in phase 1 and RPOD in phase 2), a modified version $f^{l}$ has been reported to improve convergence of the GA. [45],

(10) $$f^{l}=\log\;\left(f+\epsilon \right)$$

being *ϵ* a very small value ensuring the logarithm’s existence.

### 2.3. Phase 1: Modelization of Torsional Wave Propagation in Tissue

Phase 1 aims at checking the suitability of the propagation model and the inverse problem for optimization purposes with torsional waves. Additionally, the POD estimator is implemented and tested. Table 1 shows the steps followed in this phase.

In order to show the capability of torsional waves to characterize changes in mechanical properties of soft tissue, a direct contact emitter-receiver configuration on a layered tissue with parallel interfaces is hypothesized (see Figure 1). Torsional waves are generated and propagated through the normal direction to the layer interfaces and are collected on the opposite face of the tissue. Each layer is assumed to be isotropic. A total tissue of 40 mm with four layers of the same thickness will be assumed in this study.

**Figure 3:**
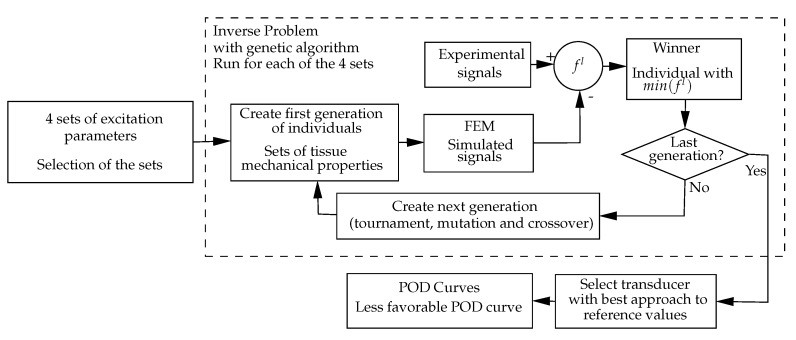
Calculation method for phase 1.

Several important points are remarked as a summary,

- A linear elastic, attenuating and multilayered physical model, solved by finite elements, was used to simulate the torsional wave propagation.
- An inverse problem is proposed to characterize the mechanical properties of the tissue and detect pathology.
- The inverse problem is applied to several sets of excitation parameters (geometry and emitted waveforms), to see the capability of the method to select that with best detection.
- The use of the semi-analytic POD estimator with the selected excitation, to measure its capability of detection.
- The calculation process is outlined in Figure 3.

Step 1 defines the problem and the four sets of excitation parameters. The adopted ranges of variation of the parameters are a frequency (*f*) range of (2,20) kHz, bandwidth (*b*) of (0.5f,f) and a transducer radius (*r*) range of (10,20) mm.

Within the mentioned ranges, four arbitrary candidates are shown in Table 2.

Following with steps 2 and 3, the torsional propagation process is described using a 3D physical model consisting of standard linear elastic equations with Rayleigh attenuation. Equilibrium, constitutive and kinematic equations are,

(11) $$\begin{array}{c} \sigma_{ji,j}+b_{i}=\rho \ddot{u_{i}}+R\rho {\dot{u}}_{i}\;\;\left\{\begin{array}{c} \sigma_{ij}=c_{ijkl}\varepsilon_{kl}\\ \varepsilon_{kl}=\frac{1}{2}\left(u_{k,l}+u_{l,k}\right) \end{array}\right. \end{array}$$

being $\sigma_{ij}$ the stress tensor , $\varepsilon_{ij}$ the strain tensor, $u_{i}$ the displacement vector, $b_{i}$ volumetric forces, *ρ* material’s density, *R* the Rayleigh damping coefficient, *C_ijkl_* the fourth-order stiffness tensor of material properties.

These equations are applied over every layer of the tissue considering continuity on displacement and stress through the interfaces. Every layer is supposed homogeneous and isotropic. In the case hereby treated, four layers of soft tissue with different mechanical properties are assumed for algorithmic testing purposes. The first and last layers are assumed to be the same tissue features, as it use to happen in many experiences, for instance, with epithelial tissue in emission and reception. In such conditions, the parameters **P** of the model are the mechanical properties of every layer, in this case, their Young moduli *E* and Rayleigh attenuation *R*, which can be presented in the vector $P=(E_{4}R_{4}E_{3}R_{3}E_{2}R_{2}E_{1}R_{1})$, being in this case $N_{p}=8$, the number of parameters. But a simpler parametrization will be used to improve IP convergence using $N_{p}=4$ parameters, $p=(E_{3}R_{3}E_{2}R_{2})$, assuming the rest of the parameters constant, as indicated in Table 3.

The forward problem is performed using a 3D FEM model implemented in FEAP package [46]. Solid hexaedric elements with 8 nodes are used in 26 blocks to generate a structured mesh (Figure 1), so that remeshing perturbation is avoided on sensitivity analysis. The material’s diameter being simulated is 100 mm. Other FEM parameters: total time 3000 μs, incremental time 20 μs and mesh extension (radius) 50 mm.

Summarizing, the model system is defined by the specimen materials (steel and four layers of tissue, Table 3), geometry and the boundary conditions: at the cylindrical axis, r = 0 mm (being r the radial direction), null displacements at the points; energy absorbing face at the external face at r = 100 mm to avoid reflections; and free displacements at the rest of the mesh points. The input is introduced as an applied displacement with the shape of a spike pulse.

Two criteria have been tested to verify the validity of the FEM model. First, a mesh convergence test has been carried out, introducing a trade-off between computational cost and numerical precision. Second, a verification of compressional (*p*) and shear (*s*) wave speeds have been compared to the time of arrival of the first wavefront.

In the second criteria, the speed of compressional $c_{p}$ and shear $c_{s}$ waves are estimated, in relationship to the bulk modulus *K* of all layers (and consequently to Young’s modulus *E*) by,

(12) $$K=\frac{E}{3(1-2\nu )}=\rho \left(c_{p}^{2}-\frac{4}{3}c_{s}^{2}\right)$$

(13) $$c_{p}=\sqrt{\frac{E(1-\nu )}{\rho (1+\nu )(1-2\nu )}}=\sqrt{\frac{K+\frac{4}{3}G}{\rho }}=\sqrt{\frac{M}{\rho }}\;c_{s}=\sqrt{\frac{G}{\rho }}$$

In particular, for $\rho =1070\;\mathrm{kg}/m^{3}$, E=20MPa and ν=0.3, $t_{p}=0.6\;\mu s$ and $t_{s}=1.1\;\mu s$ is the time used for compression and shear waves to propagate with speeds $c_{s}=0.05∼0.5\;\mathrm{km}/s$ and $c_{p}=0.94∼1\;\mathrm{km}/s$. For ν=0.49, the corresponding times are $t_{p}=0.25\;\mu s$ and $t_{s}=1.16\;\mu s$. If $\rho =1000\;\mathrm{kg}/m^{3}$ and $c_{p}=1.5\;\mathrm{km}/s$, M=2.25GPa and estimation of total time is t=1.46μs for maximum time of propagation of waves across the soft tissue.

For the inverse problem calculation, shown in Figure 3, let $\psi^{0}$ be the samples of the received signals in the case of healthy tissue, $\psi^{x}$ the samples of the received signal of the test with the soft tissue on its unknown current state, and *ψ* the samples of the simulated signal on reception. The discrepancy against the healthy state may initially be described by,

(14) $$\Phi =\frac{\psi -\psi^{0}}{\mathrm{RMS}(\psi^{0})}$$

The misfit or discrepancy between simulated and experimental signals is defined as the residual $\gamma =\Phi^{x}-\Phi$. The definition of the fitness function *f* is generated as a quadratic form from the residual vector *γ* of size *M*,

(15) $$f=\frac{1}{2}{\left|\gamma \right|}^{2}=\frac{1}{2}\frac{1}{M}\sum_{j=1}^{M}\gamma_{j}^{2}$$

Synthetic signals are created as experimental signals. Four received signals are calculated using the FEM model, one for each of the four set of excitation parameters. In order to transform these signals, avoiding exact matching between experiment and simulation, random Gaussian white noise is added (same function for all signals).

Finally, for POD estimation of the best excitation conditions, four curves will be obtained, one for each parameter of the vector **p**. The less favorable is considered.

### 2.4. Phase 2: Tissue-Transducer Modelization, Optimization with RPOD

**Figure 4:**
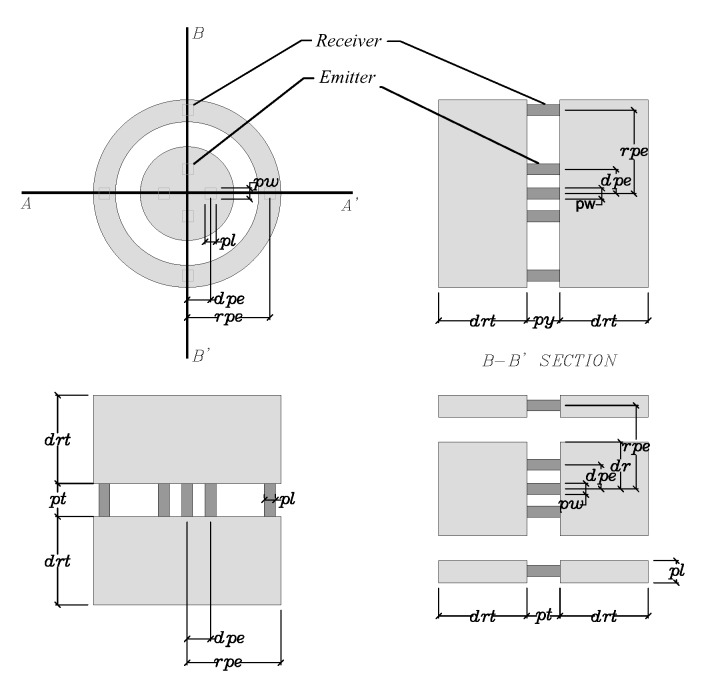
Transducer geometry. Piezoelectric elements in dark gray. Emitters at the inner disk.

The conceptual transducer design to be optimized is shown in Figure 2 and Figure 4. A central inner disk with four piezoelectric emitters electrically joined, create a circumferential shear displacement of the disk, which is transmitted to a thin double-layered tissue (dermic and connective, Figure 2, bottom area), and from it to the external circular crown where four piezoelectric elements captures the shear movement generating one signal. The time of flight is measured to infer shear moduli of the tissue layers.

The shear moduli of the layers $P=(G_{c}\;G_{d})$ (*c* connective, *d* dermic) vary when pathologic changes occur, $p=\left(\Delta G_{c}\;\Delta G_{d}\right)$, $\Delta G_{k}=G_{k}-G_{k}^{r}$, k=c,d, and this change must be greater than noise level.

Table 4 summarizes the methodology of this phase.

The forward model (Step 1) includes the coupled electrical-mechanical piezoelectric effect, under the following constitutive equations,

(16) $$\begin{array}{cc} T= & C_{E}\cdot S+e^{T}\cdot E \end{array}$$

(17) $$\begin{array}{cc} D= & e\cdot S-\varepsilon_{S}\cdot E \end{array}$$

being **T** the stress tensor, **S** strain tensor, **E** electric field, **D** charge-density displacement, $C_{E}$ piezoelectric stiffness matrix, **e** piezoelectric coupling coefficient matrix, $e^{T}$ its transpose and $\varepsilon_{S}$ permittivity coefficient matrix.

Equilibrium and kinematic relationships are as follows,

(18) $$\begin{array}{ccc} \nabla \cdot D= & 0;\;E= & -\nabla \phi \end{array}$$

(19) $$\begin{array}{ccc} \nabla^{S}\cdot T= & 0;\;S= & \frac{1}{2}\left(\nabla u+\nabla u^{T}\right) \end{array}$$

with *ϕ* the electric potential and $u=(u_{1}u_{2}u_{3})$ the vector field of displacements.

FEAP software is used to solve the FEM algorithm. The mesh and the stress distribution at different instants can be seen in Figure 2 (see [31] for more detail).

The transducer parameters represents and an 8 dimensional space (*pw, pl, pt, dpe, dr, rpe, rw, drt*) as depicted in Figure 4. A sensitivity analysis of this parameters determines their range of variation that will be used in next steps. Additionally, another sensitivity analysis of the parameters$\left(G_{d}^{r}G_{c}^{r}\right)$ on the POD is performed for values 0.3, 3 and 30 kPa, which is shown in Figure 6.

The RPOD optimization runs an inverse problem that searches for the optimal transducer parameters that maximize the RPOD. The RPOD calculation considers a 2-dimensional space of tissue mechanical parameters of reference $\left(G_{d}^{r}G_{c}^{r}\right)$ to explore a wide range of possible tissues.

Figure 7 shows the schematics of the GA, being Equation (9) the optimization criterion. A generation of $N_{d}=20$ specific individuals (transducers designs) from the 8D space are firstly selected. For each individual *d*, the parameters ${\left[S_{kj}\right]}_{d}$ (Equation (6)) are calculated for $N_{j}=10$ samples of the 2D space $\left(G_{d}^{r}G_{c}^{r}\right)$, selected using a Montecarlo procedure, obtaining $2xN_{j}$ values of $S_{kj}$ . Then, the values of ${\left[\frac{p_{kj}}{\sigma }\right]}_{d}$ (see Equation (9)) are calculated for a fixed desired level of 99.9% POD. The minimum value ${\left[\frac{p}{\sigma }\right]}_{d}$ is selected, thus implementing the Robust POD idea (RPOD), where any other values of reference $\left(G_{d}^{r}G_{c}^{r}\right)$ for the individual *d* will have a better POD. So, among all individuals of the generation, $d=1\ldots N_{d}$, that with maximum RPOD is selected (higher value $\left[\frac{p}{\sigma }\right]$). A new generation is formed combining the features of the transducers from the first generation with operations of tournament, mutation, and cross-over [44] and the process is repeated until 50th generation has been calculated. The overall winner, is the transducer design with optimized capacity of detection.

## 3. Results

Beginning with phase 1, Table 5, shows the optimized mechanical properties that corresponds to the minimized misfit function(discrepancy) between experimental (synthetic) signals and simulated signals, for each of the 4 sets of excitation parameters. Design 3 presents the better minimization of the fitness function, since is the one that best approaches the reference values of the mechanical properties.

Figure 8a shows the POD estimation of the selected Design Number 3 for increasing degraded values for a fixed noise level 10%. There are four curves of cumulative probability, one for each parameter $p_{k}$. Initially, the less favourable curve would be the one with higher displacement to the right, but taking relative variations instead of absolute values, it results in $E_{2}$, so it is conservatively taken as the one which determines the probability of detection. The convergence evolution through generations for Design number 1 is shown in Figure 8b.

The POD curves show that the properties of the second layer can be detected to a level of $\Delta E_{2}=0.5$ MPa (9.12% of the reference value of the property) and $\Delta R_{2}=100$ (4.47% respectively) with a probability above 99%, under the simulated noise. Similarly, properties of third layer $\Delta E_{3}=1$ MPa (3.65% of the reference value) and $\Delta R_{3}=100$ (4.47%) are detectable with probability above 99%. These figures are compatible with the detectability based on the global minima of the fitness function (with results in Table 5, Design 3). In a conservative position, the POD curve of the selected design in the presented situation can be determined by that of the parameter of $E_{2}$, that is the less favorable curve.

The excitation of Design 3 has been found to provide the higher sensitivity to pathology and, therefore, accurate identifiability, where a frequency range between 0.2 and 2 MHz delivers stable results.

**Figure 5:**
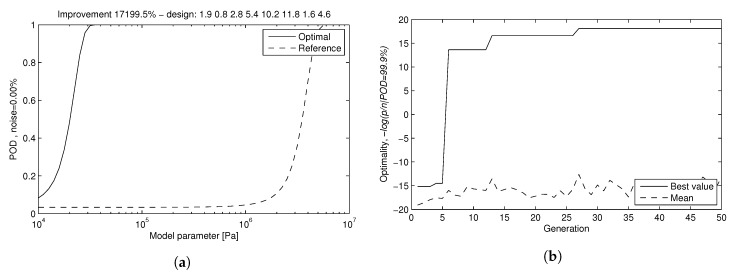
Result of the RPOD optimization process in phase 2. (**a**) RPOD functions of the optimized transducer design and the reference design; (**b**) evolution through generations; best design captured in 27th generation.

**Figure 6:**
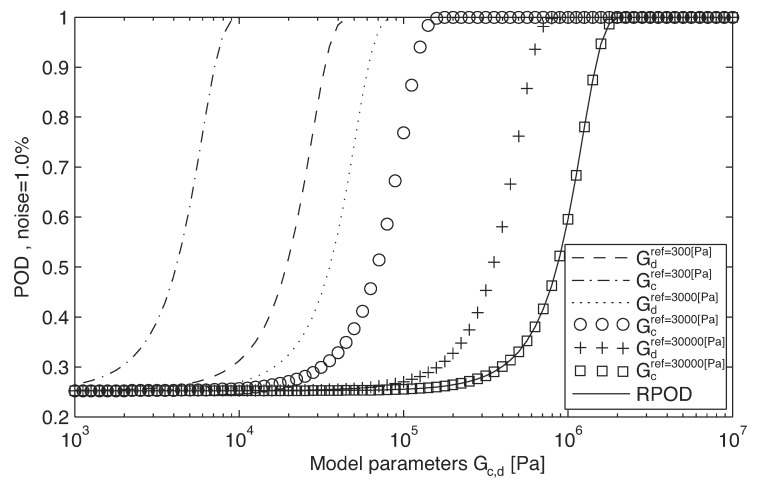
RPOD evaluation (Step 7) of phase 2, for different shear modulus values of reference.

**Figure 7:**
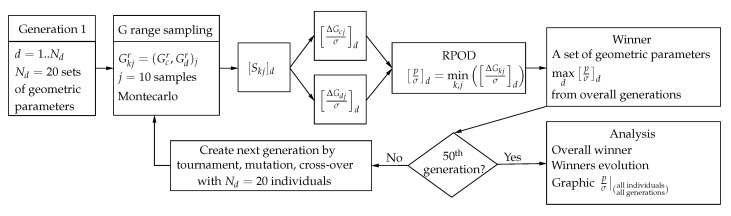
Optimization process maximizing RPOD with the genetic algorithm.

**Figure 8:**
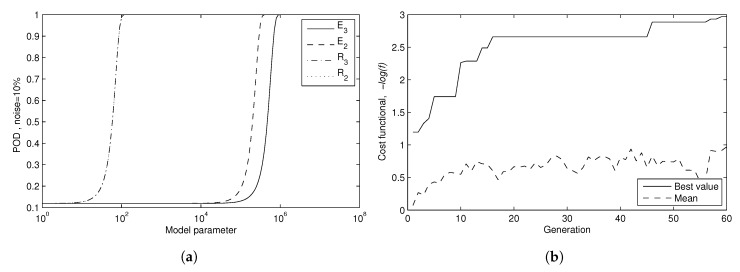
Results of phase 1. (**a**) POD dependency on mechanical properties; (**b**) convergence evolution of Design 1.

The second phase presents the following results: (a) the optimized transducer with the best geometry (winner), shown in Table 6, that maximizes RPOD, applied on the less favorable values of shear moduli of the reference; (b) the evolution of the convergence process through the generations, in Figure 5b; (c) the sampled function of eight dimensions $\frac{p_{k}}{\sigma }\left(pw,pl,pt,dpe,dr,rpe,rw,drt\right)$ used as the criterion decision in the maximization process.

Figure 5a shows the RPOD function of the winner transducer and the initial of the reference. The optimization presents a improvement of 17,199.5% (172-times better) in sensibility (in RPOD), detecting lower changes in the mechanical properties.

The configuration of the optimized transducer produces an excitation with a central frequency of 28 kHz.

## 4. Discussion and Conclusions

A strategy to optimize the design parameters of a torsional shear ultrasonic transducer, based on the inverse problem, has been developed. The main points of the methodology are a forward procedure based on FEM and analytical simplified validation with a sensibility analysis, and an inverse procedure to maximize the transducer sensitivity using a POD estimator which is developed in a robust manner to improve outcomes. Previously, in the first phase a similar methodology is performed to study just the propagation of torsional shear waves across the soft tissue. The parameters of the excitation waves and dimensions has been adjusted looking for the maximum reduction of undesired P wave emissions. The results obtained in both cases are the design parameters and biomechanical clear identification of the mechanical properties of the soft tissue layers within the expected ranges of variation.

This general methodology of a transducer optimization based on maximizing the probability of detection, together with the use of torsional waves, can open an additional line of research to obtain transducers for soft tissue evaluation at low levels of acoustic energy.

In a second step, the desired geometry of the transducer has been manufactured and currently facing adjustments of its functional performance. In the following work, this torsional ultrasonic design will be experimentally validated and improved characterizing mechanical properties in quasi-fluids and soft tissues. The experimental setup will be performed using tissue mimicking phantoms proving a sensor sensitivity study (in terms of angles of incidence and pressures) assessing the robustness of a new proposed elastography technique based on torsional quantitative principles. Three rheological models will be fitted with the experimental data beside a static independent testing method. The results of the previous rheological tests will be compared with the transducer reconstruction of the biomechanical parameters from the propagated torsional wave.

The transducer manufacturing is presenting the following challenges. The material of the ring, the steel, has been changed to polylactic acid (PLA) to obtain a better amplitude of the received wave, the same occurs with the emitter disk that has been replaced by a small motor with a PLA disk in the contact surface. The whole analytical modelization is still under investigation due to the complexity to make the problem tractable, although this is not affecting the functionality of the transducer. On the contrary, minimization of mechanical and electronic cross-talk effects between emission and reception are being faced before proceeding to the experimental validation.

Safer tests based on ultrasonic techniques than those using ionizing radiations are being investigated and some good results have been arisen, like those of Yonetsu ([47]) using quantitative sonography, showing a capacity to characterize tumors and differentiate their benign and malignant nature. As changes in consistency of tumors alters their mechanics, mechanical waves could represent an impacting method of diagnosis because of its direct nature of detection.

## Figures and Tables

**Table 1:** Methodology of phase 1.

Step	Target	Outcome	Tools or Inputs
1. Problem configuration	Problem geometry, boundary conditions, configuration, sets of excitation parameters	Problem definition. 4 sets of excitation parameters to test	
2. Forward physical model selection	Simulate propagation with the excitation parameters	Physical model: differential equations	Outcome from step 1: geometry, materials, etc.
3. Finite element model (FEM)	Computational implementation of the physical model	Computational code of the model	Outcomes from steps 1 and 2.
4. Discretization study and FEM test	Convergence study Balance computational burden-time Assure accuracy of simulations	Spatial element Time interval FEM checked	Forward model (FEM) Geometrical parameters and tissue mechanical properties L-S wave speed check
5. Inverse problem with genetic algorithm (GA)	Design and implementation Evaluate GA’s convergence - Quality of identification - Convergence speed Apply IP to 4 excitation parameter sets	Observed right behaviour and identification on the mechanical properties of the tissue Winner: excitation parameter set with best identification	FEM Cost functional for GA optimization: misfit function Synthetic signals
6. POD evaluation of the winner	POD estimator evaluation Checking coherency on results	Graphics of POD on modifications of mechanical parameters of tissue No perceived anomalies	Forward model Winner parameter set POD estimator

**Table 2:** Sets of excitation parameters under test.

Transducer	*f* (kHz)	*b* (kHz)	*r* (mm)
Design 1	6.32	6.32	10
Design 2	20	20	10
Design 3	2	1	10
Design 4	6.32	3.16	20

**Table 3:** Parameters of the layered soft tissue. Poisson’s ratio depends on *E*.

Material	Young Modulus *E* (MPa)	Poisson Ratio *ν*	Density *ρ* (kg/m^3^)	Thickness *a* (mm)	Attenuation $R\;(\frac{\mathrm{dB}}{\mathrm{MHz}\cdot \mathrm{mm}})$
Layer 1	20	0.48	1070	10	1836.07
Layer 2	$E_{2}\in \left[2∼15\right]$	$\nu_{2}$	920	10	$R_{2}\in \left[1∼5\right]$
Layer 3	$E_{3}\in \left[15∼50\right]$	$\nu_{3}$	1070	10	$R_{3}\in \left[1∼5\right]$
Layer 4	20	0.48	1070	10	1836.07

**Table 4:** Methodology of phase 2.

Step	Target	Results	Tools or Inputs
1. Selection of the forward model	Simulate generation, propagation, and reception of waves	Physical model: differential equations	Initial transducer design: geometry, materials, etc.
2. Finite element model (FEM)	Computational implementation of the physical model	Computational code of the model	Physical model Boundary conditions(geometry and properties of conceptual design, etc.)
3. Discretization analysis	Convergence enhancement Balance computational burden-time	Spatial element Time interval	FEM Initial geometry and mechanical properties
4. Validation of FEM results	Check accuracy on simulations	Test discrepancies using simplified models	Approximated analytic model of torsional waves for comparison ([31]).
5. Sensitivity test on mechanical properties of tissue and parameters of the transducer geometry ([31])	To know the response to transducer geometry and tissue propagation Select materials Reduce P-wave generation and propagation Maximize amplitude of received torsional wave New check of the FEM	Material influence Select a transducer geometry among tested vanishing P-wave Ranges of mechanical properties in tissue with low P-wave propagation Ranges of P-wave existence	FEM Materials to test Variation ranges of the transducer parameters (geometry)
6. Inverse problem as a new test of the forward problem	New check of the FEM Quality of identification of tissue mechanical properties	Valid identification of mechanical properties $\left(G_{c}\;G_{d}\right)$ of the tissue (Figure 5)	FEM Cost function for GA optimization: Misfit function
7. Evaluation of RPOD for best sensor in step 5 ([31])	Test RPOD estimator Evaluate POD for three values of tissue shear modulus of reference (0.3, 3 and 30 kPa) Testing coherency on results	POD graphics (Figure 6) on a range of values of $\left(G_{c}^{r},\;G_{d}^{r}\right)$ Select the initial parameters for optimization No apparent anomalies	FEM POD estimator
8. Transducer optimization with best RPOD criterion	Find transducer design (geometry) with maximum RPOD Evaluate improvement in RPOD	Select the optimal geometric design with the worst response to mechanical properties Quantification of improvement	FEM RPOD estimator GA design with RPOD as function to maximize

**Table 5:** Adjusted parameter for each set of excitation conditions including the relative error against reference values. Thus, Number 3 is the optimal design.

IP Results	$E_{3}$ (MPa)	$E_{2}$ (MPa)	$R_{3}\;(10^{-3})$	$R_{2}\;{(10}^{-3})$	*f* (kHz)	*b* (kHz)	*rs* (mm)
Reference Values	27.3861	5.4772	2.2361	2.2361			
Design 1	30.4330 (11%)	5.3381 (3%)	1.6567 (26%)	3.3893 (52%)	6.32	6.32	10
Design 2	25.2412 (8%)	5.9172 (8%)	1.7507 (22%)	3.2839 (47%)	20	20	10
Design 3	29.1313 (6%)	5.1269 (6%)	2.4648 (10%)	1.7845 (20%)	2	1	10
Design 4	30.6323 (12%)	5.0484 (8%)	2.5155 (12%)	1.7478 (22%)	6.32	3.16	20

**Table 6:** Optimized transducer, along with assumed ranges and the initial values of reference.

Design Parameters (mm)	Range	Initial Value	Optimal Value	Label
Piezoelectric Length	(0.5, 2)	1	0.8	pl
Piezoelectric Width	(0.75, 2)	1	1.9	pw
Piezoelectric Thickness	(0.4, 4)	2	2.8	pt
Disc Radius	(1.75, 5.75)	4.25	5.1	dr
Disc Piezoelectric Eccentricity	(1.5, 3.5)	2.5	2.7	dpe
Ring Width	(1.5, 2.5)	2	1.6	rw
Ring Piezoelectric Eccentricity	(5.75, 8.5)	7.5	5.9	rpe
Disc-Ring Thickness	(3, 13)	8	4.6	drt

## Supplementary Materials

The following are available online at http://www.mdpi.com/1424-8220/17/6/1402/s1.
